# Higher Medical Education

**Published:** 1896-06

**Authors:** 


### Higher Medical Education.

Dr. R. B. Barron, of Macon, delivered the "Orator's Address." He dwelt upon and pleaded for higher medical education. Relative to practicing medicine purely for money, he said that money with the true physcian was a secondary consideration; that the real incentive for the best work with those practitioners who achieve success was that broad humanitarianism which impelled with resistless force the commissioned agents of God Almighty to relieve the suffering, to stay the pangs of agonizing pain, to fight to the bitter end humanity's implacable and unconquerable enemy—death—regardless of any other consideration.

